# Descriptive cohort study of transient isolated tubular acidosis in early childhood: experience from a rare kidney disease expert center

**DOI:** 10.1007/s00431-025-06394-0

**Published:** 2025-09-12

**Authors:** Hajer Charfi, Aurelia Bertholet-Thomas, Justine Bacchetta, Sacha Flammier, Laurence Derain Dubourg, Aurélie De Mul

**Affiliations:** 1 Centre de Référence Des Maladies Rénales Rares, Centre de Référence Des Maladies Rares du Calcium Et du Phosphore, Filières Maladies Rares ORKiD, OSCAR Et ERKNet, Hôpital Femme Mère Enfant, 69677 Bron, France; 2 INSERM UMR 1033 LYOS, 69008 Lyon, France; 3 Faculté de Médecine Lyon Est, Université de Lyon, Lyon, France; 4 Service de Néphrologie, Dialyse, Hypertension Et Exploration Fonctionnelle Rénale, Centre de Référence Des Maladies Rénales Rares MAREGE, Filière Maladies Rares ORKID, Hôpital E. Herriot, Hospices Civils de Lyon, Lyon, France; 5 CNRS UMR 5305, University of Lyon 1, Lyon, France; 6 INSERM CARMEN 1060 IRIS Team, Lyon, France

**Keywords:** Tubular acidosis, Linear growth faltering, Failure to thrive, Bicarbonate, Sodium bicarbonate supplementation

## Abstract

Growth faltering and/or failure to thrive in children often prompts metabolic assessment, sometimes revealing metabolic acidosis and leading to referral to pediatric nephrology. Renal tubular acidosis (RTA), causing hyperchloremic metabolic acidosis due to impaired renal acidification, includes transient isolated RTA, a relatively frequent but poorly described condition. We reviewed pediatric patients referred to the Lyon Rare Kidney Disease Center (MAREGE) between March 2023 and March 2024 for linear growth faltering and/or failure to thrive associated with isolated metabolic acidosis, after excluding systemic, endocrine, and gastrointestinal causes. Patients with suspected secondary, genetic forms, or distal RTA were excluded. Follow-up was analyzed from the initial nephrology consultation to the last visit before March 2025. Data are presented as medians [IQR] and compared using non-parametric tests. Thirty-five patients were included. At diagnosis, age was 15.3 [13.1–25.6] months, with weight and height standard deviation (SD) scores of − 1.5 [− 2.5; − 1.0] and − 1.0 [− 2.0; 0.0], respectively. Tubular assessments showed low plasma bicarbonate (19 [18–20] mmol/L), non-adapted bicarbonaturia (8.0 [2.8–18.6] mmol/L), and elevated urinary pH (7.0 [6.4–7.4]). After a follow-up of 2.2 [1.4–3.1] years at an of age 3.4 [1.9–4.1] years, weight SD scores increased significantly (− 1.0 [− 1.9; − 0.4], *p* = 0.04). Height SD scores also increased (− 0.5 [− 1.5; 0.0]), though not significantly (*p* = 0.41). Catch-up growth in weight, height, or both was achieved in 77% of patients. Bicarbonate supplementation, initiated at 1.2 [0.7–1.6] mmol/kg/day, was discontinued in 54% of cases; for others, dosing remained stable (1.3 [0.9–2.0] mmol/kg/day, *p* = 0.16).

*Conclusion*: Transient isolated RTA is observed in infants and young children with mild metabolic acidosis, isolated bicarbonaturia, and moderate failure to thrive and/or growth faltering. It resolves spontaneously within a few years, usually requiring only low-dose alkalizing therapy.

**Table:** 

**What is Known:**
• *Renal tubular acidosis (RTA) is a hyperchloremic, normal anion gap metabolic acidosis due to impaired renal acidification. In children, it is a recognized cause of growth faltering or failure to thrive and is usually a genetic disease (either proximal or distal acidosis).*
**What is New:**
•* In cases of mild metabolic acidosis associated with moderate growth delay, without other tubular or extrarenal abnormalities, the possibility of transient isolated renal tubular acidosis should be considered. This condition warrants parental reassurance, as most patients show catch-up growth with minimal bicarbonate supplementation, which can usually be discontinued over time.*

## Introduction

Renal tubular acidosis (RTA) is characterized by hyperchloremic normal anion gap metabolic acidosis secondary to abnormalities of renal acidification [1, 2]. Isolated proximal renal tubular acidosis (pRTA, type II) is a rare subtype caused by a defect in bicarbonate (HCO_3_^-^) reabsorption within the proximal tubule [3]. This defect lowers the reabsorption threshold for bicarbonate, exceeding the capacity of distal segments to compensate. As a result, bicarbonate is excreted in the urine once plasma concentrations exceed a specific threshold (typically 16–20 mmol/L). As bicarbonate losses lower the plasma level below this threshold, the filtered load falls and urinary bicarbonate excretion ceases.

In isolated pRTA, there is no evidence of generalized proximal tubular dysfunction seen in Fanconi syndrome, characterized by associated hypophosphatemia, tubular proteinuria, hypouricemia, and euglycemic glycosuria [3, 4]. Moreover, clinical signs typically associated with distal renal tubular acidosis, such as nephrocalcinosis, are absent [5]. Furthermore, urinary (U) calcium and citrate levels remain within normal ranges, while UpH can be low (< 5.5) when patients are below their bicarbonate threshold due to intact distal acidification processes. Clinically, children typically present with linear growth faltering and/or failure to thrive.

Several transporters have been implicated, most notably the basolateral sodium–bicarbonate cotransporter NBCe1, the apical sodium–proton exchanger NHE3, and the H^+^‑ATPase [3, 6]. Isolated pRTA can be inherited in either an autosomal‑recessive or autosomal‑dominant manner or acquired after exposure to carbonic anhydrase inhibitors. The autosomal recessive form, first described by Igarashi et al., is linked to mutations in the *SLC4A4* gene, encoding the NBCe1 cotransporter [7, 8]. This variant is associated with severe growth retardation, ocular abnormalities (including glaucoma, cataracts, and band keratopathy), and intellectual disability. Additionally, pancreatic involvement and calcifications in the basal ganglia have been documented. Affected patients exhibit profound metabolic acidosis (bicarbonate levels usually below 15 mmol/L) and require high doses of bicarbonate supplementation, typically ranging from 5 to 15 mmol/kg/day [7, 8]. In contrast, two families have been reported with isolated pRTA following an autosomal dominant pattern of inheritance, accompanied by reduced bone density on densitometry studies [9, 10]. However, despite extensive genetic analysis, the precise molecular basis remains undetermined.

Poor growth, particularly affecting body weight, associated with metabolic acidosis is a frequent reason for referral to pediatric nephrology. In cases of mild isolated RTA, physicians usually attribute it to tubular immaturity. Children are given sodium bicarbonate supplementation, typically at a low daily dose, over several months or even years. Gradually, the bicarbonate supplementation is reduced and eventually stopped. This clinical description corresponds to transient isolated RTA. However, to date, only nine cases have been documented in the literature [11].

The present study aims to refine the clinical and biochemical phenotype of children with transient isolated RTA who were evaluated and followed at a national referral center for rare pediatric kidney diseases.

## Methods

### Study design

We reviewed the medical records of all patients who attended an outpatient consultation at the Rare Kidney Disease Reference Center of Lyon (MAREGE) between March 2023 and March 2024 for failure to thrive and/or linear growth faltering associated with isolated metabolic acidosis (as defined below) for initial referral or follow-up. Records were analyzed from the initial pediatric nephrology consultation through to the latest follow-up visit completed by the end of March 2025. All procedures were conducted in accordance with the Declaration of Helsinki. This study received approval from the local Institutional Review Board (IRB) (Comité d’Éthique des Hospices Civils de Lyon, N° 24–5513).

### Definition of isolated tubular acidosis

**Fig. 1:**
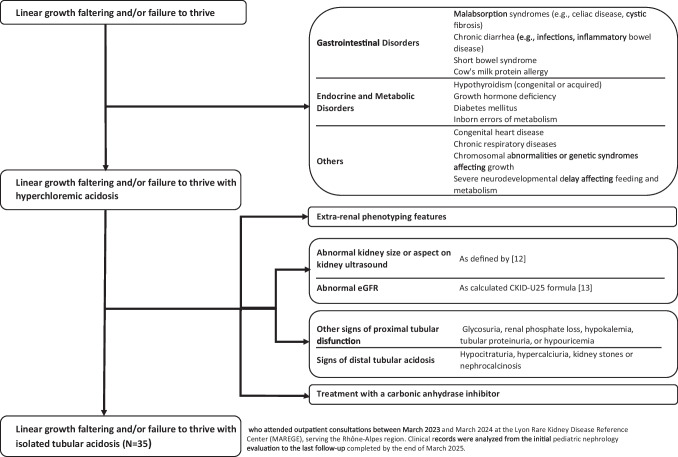
Inclusion process

We included patients with failure to thrive and/or linear growth faltering in the context of isolated metabolic acidosis only if a comprehensive work-up had already been performed to rule out systemic, endocrine, and gastrointestinal causes. Only patients with normal kidney function and size, as confirmed by estimated glomerular filtration rate (eGFR) and renal ultrasound findings, were included. Isolated metabolic acidosis was defined as hyperchloremic acidosis with normal anion gap, in a non-hemolyzed venous blood sample. The following patients with metabolic acidosis were not considered to have isolated tubular acidosis: extra-renal phenotypic features, other signs of proximal tubular dysfunction (glycosuria, renal phosphate loss, hypokalemia, tubular proteinuria, or hypouricemia), signs of distal tubular acidosis (kidney stone or nephrocalcinosis on renal ultrasound, hypocitraturia, or hypercalciuria), and treatment with a carbonic anhydrase inhibitor. Adequate acidosis control was defined as plasma or serum bicarbonate ≥ 22.0 mmol/L [5]. Figure 1 summarizes the patient inclusion process.

### Biomarkers

During a tubular function assessment conducted outside of any infectious episodes, plasma and urine samples were collected simultaneously to measure levels of sodium, potassium, glucose, bicarbonate, proteins, calcium, phosphate, magnesium, uric acid, and osmolality using standard laboratory techniques. Standardized creatinine levels were determined by isotope-dilution mass spectrometry, and the eGFR was calculated using the CKID-U25 formula [13]. The tubular reabsorption of electrolytes was calculated, along with the tubular maximum phosphate reabsorption per GFR (TmP/GFR) [14]. Additionally, urinary (U) ratios were determined, including calcium/creatinine [15], uric acid/creatinine, albumin/creatinine, β2-microglobulin/creatinine, protein/creatinine, and citrate/creatinine, as well as glycosuria. Urinary pH was measured using a specific pH electrode (Orion™ 8103 BNUWP ROSS Ultra™, Thermo Scientific). When urinary pH exceeded 6.3, urinary PCO_2_ was measured using a blood gas analyzer (ABL800, Radiometer), allowing for the calculation of urinary bicarbonate concentration via the Henderson-Hasselbalch equation. When urinary pH was below 6.3, urinary bicarbonate concentration was considered clinically negligible (< 1.9 mmol/L assuming PCO_2_ = 40 mmHg). In cases where urinary pH exceeded 8.0, bicarbonaturia was assessed using a spectrophotometric enzymatic technique based on the PEPC/MDH method (Roche cobas pro®). Plasma renin activity and aldosterone were measured by chemiluminescence on an automated ISYS IDS system calibrated according to the international standard WHO 68/356 (1.67 mU/L = 1 ng/L). The local normal reference ranges for renin and aldosterone in this age group are 2.51–35.7 ng/L and 64–859 pmol/L, respectively.

### Patient characteristics

Collected data included gestational age, birth weight and height, history of prematurity, and intrauterine growth restriction (defined as a fetal weight below the 10th percentile for gestational age according to World Health Organization charts) [16]. Information regarding breastfeeding duration, age at solid food introduction, presence of vomiting, nutritional management, and symptoms reported by parents were also recorded. Nutritional management refers to a consultation with a dietitian aimed at evaluating dietary habits and adjusting nutrient and calorie intakes as needed. Clinical parameters assessed at birth and during the follow-up included height and weight expressed in standard deviation (SD) units based on French pediatric growth charts [17]. BMI was calculated as weight in kilograms divided by height in meters squared (kg/m^2^). Failure to thrive is defined as a downward shift across two or more major percentile lines on standardized growth charts [18]. Linear growth faltering is defined as a downward shift across at least two major percentile line on height-for-age charts [18]. Catch-up growth, whether in weight or height, was defined as an upward crossing of at least one major percentile line on standardized growth charts (weight-for-age or height-for-age) [19]. Parental target height was calculated using the Tanner formula [20]. Sodium bicarbonate supplementation (mmol/kg/day) and its tolerance were evaluated at the initial consultation and subsequently every 6 months until the last follow-up.

### Statistical analysis

Results are presented as medians [interquartile range, IQR] for quantitative data and number (percentage) for qualitative data. Missing data were not imputed. Non-parametric tests were used for comparisons: the Mann–Whitney test for unpaired groups; the Wilcoxon test for paired groups. A two-sided *p*-value of < 0.05 was considered statistically significant. Statistical analyses were conducted using the R statistical software 4.3.0.

## Results

### Study population characteristics at first consultation

A total of 35 patients with linear growth faltering and/or failure to thrive, in whom systemic, gastroenterological, and endocrinological causes were excluded, and who were found to have isolated metabolic acidosis, were included. The sex ratio was 0.4 (14 boys).

The median birth weight was 3220 [2880–3490] g, and the median birth length was 48.5 [47.0–50.5] cm. Median gestational age at birth was 40 [39–40] weeks. Two patients (5.7%) were born prematurely but with appropriate weight for gestational age. These two patients were born at 31 + 5 and 34 + 6 weeks of gestation. Four patients (11.4%) were full-term but experienced intrauterine growth restriction.

The median age at the onset of linear growth faltering and/or failure to thrive was 7.9 [4.9–12.0] months, whereas the median age at diagnosis was 15.3 [13.1–25.6] months. For the 21 patients with available data, the median duration of breastfeeding was 3.0 [0.0–7.0] months, and the introduction of solid food occurred at a median age of 6.0 [5.0–7.0] months. Recurrent vomiting was reported in four children (11.4%), and the parents of 14 patients (40.0%) described reduced appetite. Thirteen children (37.1%) benefited from nutritional management. No additional symptoms were reported.

For the biometric assessment at initial presentation, the median body weight in standard deviation (SD) units was − 1.5 [− 2.5; − 1.0], and the median height in SD units was − 1.0 [− 2.0; 0.0]. Median BMI at initial presentation was 14.7 [13.8–16.0] kg/m^2^. Seventeen patients (48.6%) had a weight-for-age below − 2 SD, while 11 patients (31.4%) had a height-for-age below − 2 SD. Notably, 10 patients (28.6%) had both weight-for-age and height-for-age below − 2 SD. Among patients with height and weight above − 2 SD, nine (25.7%) exhibited failure to thrive, and five (14.3%) showed linear growth faltering. Parental target height data were available for 29 patients. Among them, six patients (17.1%) had a height more than 2 SD below their parental target height.

### Initial evaluation of tubular function

Table 1 presents the initial tubular function results (*n* = 35). All studied parameters fell within age-specific reference ranges, except for serum and U bicarbonate levels, chloremia, and U pH.

**Table 1:** Initial tubular evaluation

*N* = 35		Normal range
eGFR(mL/min/1.73 m^2^)	135 [121–151]	> 90 [13]
Creatinine (µmol/L)	21 [18–23]	15–31
Sodium (mmol/L)	138 [137–140]	138–145
Potassium (mmol/L)	4.3 [4.0–4.6]	3.4–4.7
Chlore (mmol/L)	**108 [107–109]**	98–107
Bicarbonate (mmol/L)	**19 [18–20]**	20–28
Plasma anion gap (mmol/L)	11 [10–13]	12–16
Calcium (mmol/L)	2.55 [2.49–2.64]	2.20–2.70
Phosphate (mmol/L)	1.63 [1.55–1.72]	1.38–2.19 [14]
SDS phosphate	− 0.8 [− 1.1; 0.0]	
Magnesium (mmol/L)	0.89 [0.86–0.94]	0.86–1.17
Uric acid (µmol/L)^*^	223 [188–251]	100–290
Glucose (mmol/L)	4.8 [4.4–5.5]	3.5–5.5
Total protein (g/L)	70 [67–74]	61–75
ALP (UI/L)^#^	230 ([171–262)]	156–369 [21]
U osmolarity (mOsm/l)	**418 [246–652]**	500–850
U citrate/creatinine^*^ (mmol/mmol)	0.56 [0.42–0.87]	> 0.14
U calcium/creatinine (mmol/mmol)	0.40 [0.20–0.84]	0.06–1.4 [15]
U uric acid/creatinine (mmol/mmol)	1.0 [0.8–1.2]	0.7–1.5
ACR (mg/mmol)	3.4 [1.7–4.2]	< 3
UPCR (mg/mmol)#	29.5 [24.0–41.3]	< 20
U β2M/Cr (mg/mmol)¤	36 [24–46]	6.0–40.7
TmP/GFR (mmol/L)	1.51 [1.44–1.68]	1.21–1.71 [14]
U pH	**7.0 [6.4–7.4]**	4.5–6.5
U HCO_3_^−^ (mmol/L)	**8.0 [2.8–18.6]**	Not quantifiable
Urine anion gap (mmol/L)	**59 [28–91]**	Negative urine anion gap suggestive of increased urinary ammonium excretion
Glycosuria (mmol/L)	0.2 [0.1–0.3]	< 0.5

Results expressed as medians [IQR], *ACR* albumin to creatinine ratio, *ALP* alkaline phosphatase, *β2M/Cr* β2Microglobulin-to-creatinine ratio, *eGFR* estimated glomerular filtration rate, *HCO*_*3*_^*−*^ bicarbonate, *K* potassium, *Na* sodium, *SDS* standard deviation score, *Tmp/GFR* tubular maximum for phosphate reabsorption per glomerular filtration rate, *U* urinary, *UPCR* U protein-to-creatinine ratio

¤Four missing data

^*^Six missing data

^#^Nine missing data

Bold is when the values are outside the normal range

The median serum bicarbonate level was 19 [18–20] mmol/L, with a median U pH of 7.0 [6.4–7.4] and a median U bicarbonate level of 8.0 [2.8–18.6] mmol/L, which was inappropriate with respect to circulating bicarbonate levels.

Among the patients, ten were already receiving bicarbonate supplementation at the time of the initial tubular evaluation. Their median initial serum bicarbonate level, measured prior to supplementation, was 17 [15–19] mmol/L. At the time of the first tubular work-up, these patients had a median serum bicarbonate level of 21 [19–21] mmol/L, a median urinary pH of 7.3 [6.6–7.7], and a median urinary bicarbonate concentration of 19.3 [3.3–47.3] mmol/L. These values were significantly different from those observed in patients not receiving supplementation, who had a median serum bicarbonate level of 19 [18–20] mmol/L (*p* = 0.005, Mann–Whitney test) and a median urinary bicarbonate concentration of 6.4 [2.4–13.2] mmol/L (*p* = 0.046, Mann–Whitney test). However, there was no significant difference in U pH between the two groups (6.9 [6.4–7.3] vs. 7.3 [6.6–7.7], *p* = 0.19, Mann–Whitney test).

In 26 patients, total alkaline phosphatase levels were measured and found to be within the normal range, with a median value of 230 [170–262] IU/L.

Renin and aldosterone levels were measured in 27 patients, showing a median renin level of 20 [13–40] ng/L. Thirteen patients had undetectable aldosterone levels (< 108 pmol/L), while the remaining 14 had median aldosterone levels of 247 [130–464] pmol/L.

### Follow-up

At the last follow-up, the median age was 3.4 [1.9–4.1] years with a median follow-up duration of 2.2 [1.4–3.1] years.

#### Evolution of biometric parameters

At the end of follow-up, the median SD score for body weight increased significantly to − 1.0 [− 1.9; − 0.4] compared to baseline (*p* = 0.04, Wilcoxon test). The median height SD score reached − 0.5 [− 1.5; 0.0], showing a non-significant increase compared to the initial evaluation (*p* = 0.41, Wilcoxon test). Median BMI remained stable at 14.5 [13.9–15.1] kg/m^2^ (*p* = 0.51, Wilcoxon test).

Eighteen patients (51.4%) demonstrated catch-up growth in both weight and height, whereas five patients (14.3%) showed catch-up growth in weight only and four patients (11.4%) in height only. Eight patients (22.9%) did not show catch-up growth in either weight or height. Four out of 29 patients with available parental target height data remained more than 2 SD below their target height at the end of follow-up, with no significant change from baseline (*p* = 0.73, Wilcoxon test).

**Fig. 2:**
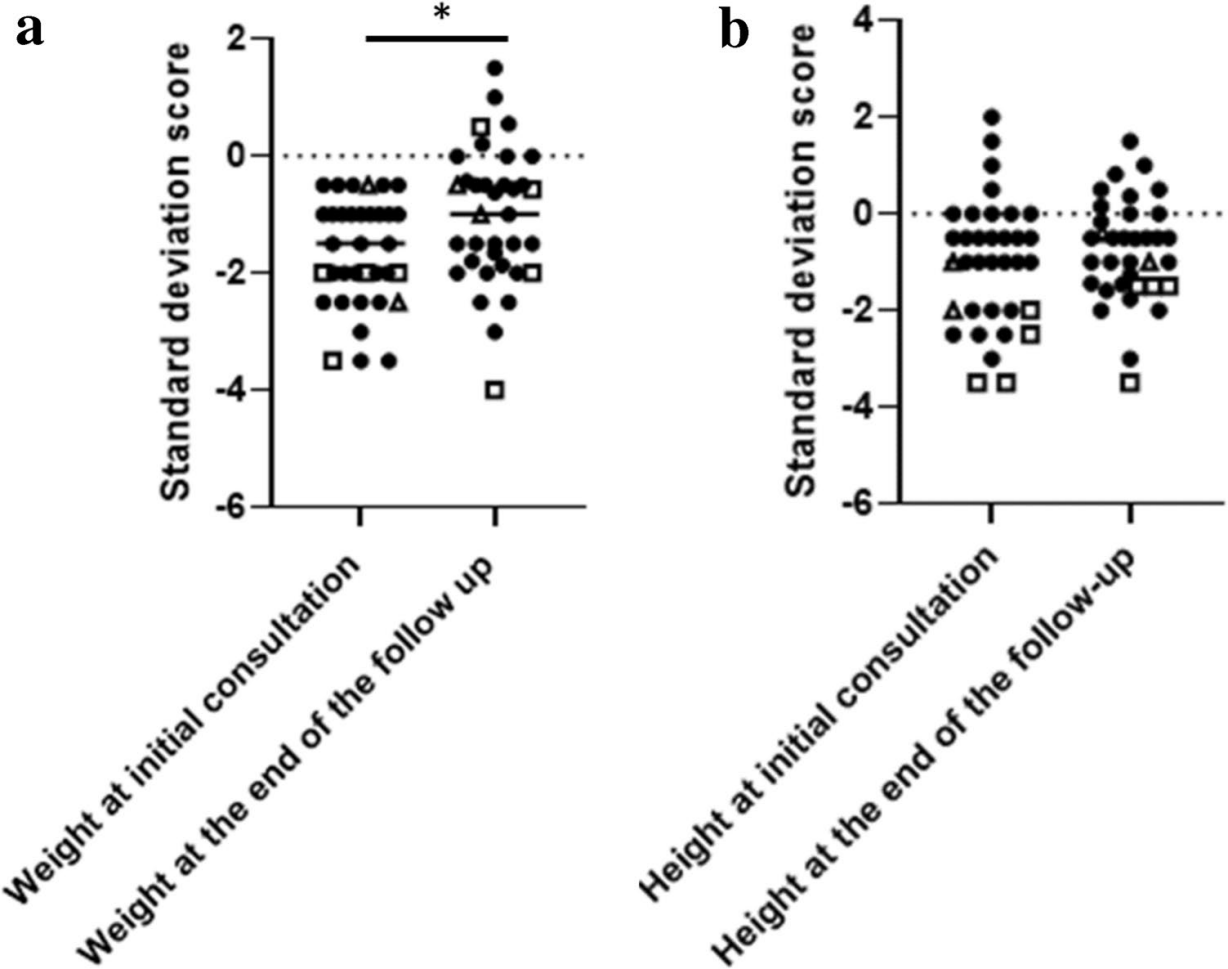
**a** Evolution of weight expressed as standard deviation scores (SDS) at baseline and at the end of follow-up. The figure shows the distribution of weight SDS. Squares represent patients with intrauterine growth restriction (IUGR), and triangles indicate those born preterm. At baseline, the median weight SDS was − 1.5 [− 2.5; − 1.0]. At the end of follow-up, weight SDS significantly improved to − 1.0 [− 1.9; − 0.4] (*p* = 0.04, Wilcoxon test). **b** Evolution of height expressed as standard deviation scores (SDS) at baseline and at the end of follow-up. The figure shows the distribution of height SDS. Squares represent patients with intrauterine growth restriction (IUGR), and triangles indicate those born preterm. At baseline, the median height SDS was − 1.0 [− 2.0; 0.0]. At the end of follow-up, height SDS showed a non-significant improvement to − 0.5 [− 1.5; 0.0] (*p* = 0.41, Wilcoxon test)

Figure 2 illustrates the evolution of SD-weight and height from baseline to the end of follow-up.

Patients who failed to show catch-up growth in both weight and height (*n* = 8) were analyzed in comparison to the rest of the study population. As shown in Table 2, no significant differences were found between the two groups (Mann–Whitney test). However, patients without catch-up were diagnosed later and had a shorter follow-up period.

**Table 2:** Comparison of patients with catch-up in at least one biometric parameter vs. non-catch-up patients

	Total cohort (*N* = 35)	Catch-up in at least one parameter (*N* = 27)	No catch-up in weight and height (*N* = 8)
IUGR or prematurity; *N* (%)	6 (17)	5 (19)	1 (13)
Birthweight (grams)	3220 [2880–3490]	3230 [2850–3530]	3200 [2978–3425]
Diagnostic age (years)	1.3 [1.1–2.1]	1.1 [1.2–1.9]	1.7 [1.3–2.8]
Onset of growth faltering and/or failure to thrive (months)	7.9 [4.9–2.0]	8.4 [4.8–12.0]	9.6 [3.6–16.8]
Initial weight (SD)	− 1.5 [− 2.5; − 1.0]	− 2.0 [− 2.5; − 1.0]	− 1.0 [− 2.4; − 0.6]
Initial height (SD)	− 1.0 [− 2.0; 0.0]	− 1.0 [− 2.0; − 0.5]	− 0.5 [− 0.9; 0.0]
Initial sodium bicarbonate supplementation (mmol/kg/day)	1.2 [0.7–1.6]	1.3 [0.8–1.5]	1.0 [0.6–1.7]
Initial serum bicarbonate level (mmol/l)	19 [18–20]	19 [18–20]	19 [18–20]
Initial urinary pH	7.0 [6.4–7.4]	6.9 [6.4–7.4]	7.2 [6.8–7.3]
Initial urinary bicarbonate (mmol/l)	8.0 [2.8–18.6]	8.0 [3.0–21.0]	7.2 [2.7–16.0]
Initial urinary Na/K	0.7 [0.4–1.1]	0.7 [0.4–1.3]	0.8 [0.5–1.0]
Serum bicarbonate level at the end of the follow-up (mmol/l)	22 [21–22]	22 [21–22]	22 [21–24]
Bicarbonate supplementation discontinued; *N* (%)	19 (55)	15 (56)	4 (50)
Follow-up duration (years)	2.2 [1.4–3.1]	2.3 [1.4–3.4]	1.7 [0.7–2.8]

Results are expressed as medians [IQR] or as number (percentage). No significant differences (Mann–Whitney test), *IUGR* intra uterine growth restriction, *N* number, *Na* sodium, *K* potassium, *SD* standard deviation

#### Evolution of tubular function parameters

By the end of follow-up, the median bicarbonate level was 22 [21–22] mmol/L, significantly higher than at the initial evaluation (*p* < 0.0001, Wilcoxon test); moreover, 19 patients (54.3%) achieved serum bicarbonate levels above 22.0 mmol/L. Twenty-seven patients underwent repeated tubular evaluations.

#### Sodium bicarbonate supplementation

Thirty-four patients received sodium bicarbonate supplementation. The median initial sodium bicarbonate supplementation was 1.2 [0.7–1.6] mmol/kg/day. The median bicarbonate supplementation over time remained stable between 1 and 2.3 mmol/kg/day. At the end of the follow-up, 16 patients (45.7%) were still supplemented with sodium bicarbonate, with a median dose of 1.3 [0.9–2.0] mmol/kg/day. Throughout the follow-up period, no discontinuation of sodium bicarbonate supplementation due to poor tolerance or adverse effects was observed.

Table 3 illustrates the evolution of serum bicarbonate levels and sodium bicarbonate supplementation over time, along with the corresponding number of patients under follow-up and receiving treatment.

**Table 3:** Evolution of serum bicarbonate levels and bicarbonate supplementation over time, along with the corresponding number of patients under follow-up and receiving treatment

Follow-up duration (months)	Sodium bicarbonate supplementation in treated patients (mmol/kg/day)	Serum bicarbonate (mmol/L)	Number of patients under follow-up	Number of treated patients
Initiation	1.2 [0.7–1.6]	19 [18–20]	35	34
0–6	1.2 [0.7–2.0]	20 [19–22]	35	33
6–12	1.5 [1.0–2.2]	20 [19–22]	32	31
12–24	1.8 [1.5–2.5]	21 [20–23]	30	24
24–36	1.0 [0.8–3.0]	20 [19–21]	24	10
36–48	2.3 [1.3–4.4]	23 [22–24]	9	5
48–60	1.3 [1.3–1.4]	22 [21–23]	4	3
60–72	1.3 [0.6–1.9]	23 [22–24]	3	2
> 72	1.2	22 and 25	2	1

Results expressed as medians [IQR]

#### Clinical outcomes in selected cases

Initial kidney ultrasound examinations were normal in all patients. Follow-up ultrasounds, performed at the discretion of the clinician 2 years after the initiation of alkalizing therapy in two patients, revealed grade I nephrocalcinosis in one patient and nephrolithiasis in another. All patients were born at term, without evidence of IUGR or hypercalciuria, and had normal serum 25(OH) vitamin D concentrations. Genetic analyses did not reveal any pathogenic variants associated with these findings. No other genetic analyses were performed in the rest of the cohort. Supplementary Table 1 provides a description of these two patients.

Among the six patients with a history of prematurity or intrauterine growth restriction (IUGR), four were diagnosed after the median age at diagnosis observed in those without prematurity or IUGR. At baseline, all six patients had at least one anthropometric parameter (height or weight SDS) more severely impaired compared to the rest of the cohort, and five of them had both parameters below the median values observed in those without prematurity or IUGR. By the end of follow-up, all six patients maintained a weight SDS below the median of those without prematurity or IUGR, and five out of six remained shorter than the median height of the rest of the cohort. Notably, only one patient failed to achieve catch-up growth in both height and weight. Sodium bicarbonate supplementation was successfully discontinued in 50% of these patients, a proportion comparable to that observed in those without prematurity or IUGR. Additionally, four of the six patients had a follow-up duration exceeding the median duration observed in the rest of the cohort. Supplementary Table 2 provides detailed clinical and biochemical characteristics of these six patients.

## Discussion

We report on a cohort of 35 pediatric patients with isolated renal tubular acidosis (RTA), characterized by mild linear growth faltering and/or failure to thrive, and a moderately reduced bicarbonate reabsorption threshold. Clinical outcomes were favorable: serum bicarbonate levels normalized in more than half of the patients, more than half discontinued supplementation due to therapeutic success, and two-third exhibited catch-up growth in height and/or weight after a median follow-up of 2.2 years.

Transient isolated RTA, often presenting as poor growth and/or suboptimal weight gain, is a common reason for pediatric nephrology consultations. Although transient isolated RTA is often mentioned in textbooks, detailed case reports in the scientific literature are sparse, and its true prevalence remains unknown. The study by Nash et al. reported nine patients (eight boys, one girl) diagnosed with isolated RTA; some of whom appeared to follow a transient course [11].

Early onset is a hallmark shared between transient isolated RTA, hereditary isolated pRTA, and other forms of renal tubular acidosis. In our series, the median age at presentation was 7.9 months, but the median age at diagnosis was 15.3 months due to time needed to complete diagnostic work-up and exclude other causes. Similarly, Nash et al. reported that all patients presented within the first 18 months of life [11]. Linear growth faltering and/or failure to thrive during the first year of life should prompt clinicians to consider this diagnosis, warranting measurement of serum bicarbonate levels using a non-hemolyzed sample which can be challenging to obtain.

Failure to thrive was the predominant clinical feature, with a median body weight in SD units of − 1.5 [− 2.5; − 1.0]. Linear growth faltering were moderate, with a median height in SD units of − 1.0 [− 2.0; 0.0], similar to findings in autosomal dominant forms [9, 10], but contrasting with the more severe growth failure (− 4 to − 5 SD) seen in autosomal recessive cases [7, 8].Vomiting, a key symptom in Nash et al.’s [11] study, was seen in only four children in our cohort, whereas nearly half reported reduced appetite. The absence of ocular abnormalities, psychomotor delays, deafness or neurological impairments, along with the lack of familial clustering, further supports the diagnosis of isolated transient RTA.

In the transient form of isolated RTA, metabolic acidosis tends to be mild with a moderately reduced threshold for U bicarbonate reabsorption. In the Nash et al.’s cohort, this threshold ranged from 17.5 to 20 mmol/L [11]. In our cohort, the degree of acidosis was similarly moderate, with a median serum bicarbonate level of 19 [18–20] mmol/L. The estimated bicarbonate reabsorption threshold in our patients ranged from approximately 15 to 19 mmol/L. Four children showed no bicarbonaturia despite serum bicarbonate at 17–18 mmol/L, suggesting thresholds below 17 mmol/L. In contrast, the remaining children had median urinary bicarbonate of 8.4 [3.0–20.6] mmol/L when serum bicarbonate was 19 [16–21] mmol/L. In contrast, the U bicarbonate reabsorption threshold is significantly lower in both autosomal recessive and dominant forms of pRTA, with serum bicarbonate levels typically between 5 to 14 mmol/L in the recessive form [7, 8] and approximately 15 mmol/L in the dominant form [9, 10]. This intermediate phenotype in our cohort was confirmed by the low median sodium bicarbonate supplementation of 1.2 [0.7–1.6] mmol/kg/day. This dosage remained stable from baseline to the most recent follow-up. This is notably lower than the supplementation levels of 5 to 15 mmol/kg/day often required in genetic forms of pRTA [7, 8].

Since the 1970s, studies have shown a moderately reduced bicarbonate threshold in preterm, including late preterm, typically improving within the first weeks of life [22–24]. In this study, only two patients were premature (31 + 5 and 34 + 6 weeks of gestation), and four presented with intrauterine growth restriction. Similarly, the transient form of RTA, attributed to developmental immaturity, exhibited a self-limiting course in this study. In this cohort, with a median age at last follow-up of 3.4 years, approximately 55% of patients were able to discontinue alkalizing treatment after a median follow-up of 2.2 years. The longest treatment duration observed in our cohort was 7.4 years, aligning with findings by Nash et al., who reported that seven out of nine patients maintained normal serum pH and bicarbonate levels without supplementation, with follow-up durations ranging from 6 months to 8 years [11].

Management through regular pediatric nephrology outpatient clinics and sodium bicarbonate supplementation (thus supplementing patients with both bicarbonate and sodium) resulted in significant increase weight gain for affected children, though the increase in height was not statistically significant, likely due to small sample size and short follow-up. However, over two-thirds exhibited catch-up growth in height, weight, or both, and only four children remained more than 2 SD below their target height at the end of follow-up. It is plausible that the follow-up period and sample size were insufficient to demonstrate a significant effect on height, especially considering that the eight patients without catch-up in either weight or height had the shortest follow-up duration. Long-term monitoring of this cohort is essential to better evaluate the impact on overall growth and development.

A comprehensive tubular workup confirmed the isolated nature of the acidosis in all patients, and before considering this diagnosis, this evaluation is warranted to rule out other tubulopathies that may benefit from a specific more intensive management (and notably Fanconi syndrome and cystinosis [25]). Here, tubular and glomerular functions were normal, aside from the metabolic acidosis and U bicarbonate loss. Specifically, no hypocitraturia, hypercalciuria, or reductions in TmP/GFR were observed. However, the development of transient grade 1 nephrocalcinosis and nephrolithiasis in two patients prompted a reevaluation of the diagnosis of transient RTA, leading to additional assessments, including genetic studies, which yielded negative results. Genetic testing should be considered during follow-up if additional tubular abnormalities, nephrocalcinosis or nephrolithiasis, or worsening of growth retardation occur.

Our study has certain limitations related to its retrospective and descriptive design, as well as its relatively small sample size. It could have been interesting to perform an exome analysis in these patients to better describe them, but the cost/efficiency of such an approach in this clinical setting is not favorable. From an ethical point of view, it is also questionable to perform such analyses in mild and isolated RTA: it could potentially identify heterozygous variants in genes associated with RTA, but in any case that would not modify patients’ management. That said, our cohort provides valuable clinical insights to better characterize this subset of patients referred to pediatric nephrologists for growth retardation associated with isolated metabolic acidosis. Importantly, our findings offer reassurance to parents regarding the generally transient and benign nature of this condition in most cases and that appropriate treatment is linked to significant weight gain in affected children.

## Conclusion

Transient isolated tubular acidosis remains poorly documented in the literature. It typically presents with mild metabolic acidosis, isolated bicarbonaturia, and moderate linear growth faltering and/or failure to thrive, without signs of additional tubular or extrarenal dysfunction. Management typically consists of low doses of alkalizing therapy, and the condition often resolves spontaneously over time. Early identification enables adequate management and parental reassurance, as serum bicarbonate levels usually normalize within a few years.

## Data Availability

No datasets were generated or analysed during the current study.
